# The Shifting Risk of Homelessness among Persons with a Disability: Insights from a National Policy Intervention

**DOI:** 10.3390/ijerph17186512

**Published:** 2020-09-07

**Authors:** Andrew Beer, Lyrian Daniel, Emma Baker, Laurence Lester

**Affiliations:** 1 UniSA Business, University of South Australia, Adelaide 5000, Australia; laurence.lester@unisa.edu.au; 2 School of Architecture and Built Environment, The University of Adelaide, Adelaide 5005, Australia; lyrian.daniel@adelaide.edu.au (L.D.); emma.baker@adelaide.edu.au (E.B.)

**Keywords:** disability, housing, homelessness, risk

## Abstract

Persons with a disability are at a far higher risk of homelessness than those without. The economic, social and health challenges faced by disabled people are addressed, in Australia, by the recently implemented National Disability Insurance Scheme (NDIS). Using nationally representative, longitudinal household panel data, we construct the Index of Relative Homelessness Risk (IRHR) to track how the risk of homelessness for disabled persons has changed since the introduction of the NDIS. We find that, overall, fewer persons with a disability face moderate risk of homelessness but that many more face high risk. We conclude that the NDIS has not effectively protected disabled people from the risk of homelessness. We reflect on the implications of these findings for policy interventions.

## 1. Introduction

In 2018, 4.4 million Australians, almost 18 per cent of the total population, were living with a disability [1]. Persons living with a disability were overrepresented among clients accessing specialist homelessness services [2] and more likely to be unemployed [3]. These challenging financial and social circumstances leave disabled persons more likely to experience housing stress, or risk of homelessness, than those in the population without a disability. At the 2016 Census fully 116,000 persons were enumerated as homeless on Census night and some 340,000 Australians experience homelessness at some time throughout the year. Government responses to homelessness have been led by State Governments, with additional funding provided by the Australian Government and have included financial support for not-for-profit agencies to provide crisis shelters, outreach programs to assist rough sleepers and support services to enable individuals enter and sustain long-term housing. We seek to understand how a major policy intervention in disability support services over the past decade has changed the risk of homelessness in Australia.

In an earlier paper [4], we examined the risk of homelessness in Australia for people with, and without, a disability, as well as by disability type and severity of functional limitation (for the period 2001–2009). We constructed the Index of Relative Homelessness Risk (IRHR) to enable consistent comparison for population subgroups. The key finding in that paper was that, overall, persons with a disability have greater exposure to the risk of homelessness, although the risk is unevenly distributed. Persons with more severe limitations, intellectual or psychological disabilities, or schooling/employment restrictions, were at higher risk of homelessness. We suggested these results were, at least partially, reflective of the welfare system in Australia; that is, that assistance was (and continues to be) unevenly targeted, provided, or taken up across the population.

Following the establishment of these baseline results, we now have the opportunity to conduct a quasi-experiment by constructing the same IRHR for disabled and non-disabled populations in the period since the rollout of the National Disability Insurance Scheme (NDIS)—Australia’s primary vehicle for supporting persons living with a disability. The analysis presented in this paper provides a second point-in-time application of the IRHR, allowing us to monitor change and form conclusions on the impact of the NDIS on the risk of homelessness for disabled persons in Australia.

### The National Disability Insurance Scheme (NDIS)

Australia’s NDIS was established “to provide reasonable and necessary supports to people with a permanent and significant disability” [5]. The NDIS was introduced gradually from 2016, and it is anticipated that by 2025 it will provide $A22 billion in support annually to approximately 500,000 Australians living with a disability. The NDIS was developed to target those Australians who acquired a permanent disability before the age of 65, which substantially reduced their capacity to manage everyday activities. Importantly, the definition of “disability” adopted by the NDIS in determining participation in the scheme is narrower than that taken be the United Nations Convention on the Rights of Persons with Disabilities, focusing on “*the reduction or loss of an ability to perform an activity* which results from an impairment” (emphasis in original) [5]. The NDIS did, however, respond to the obligations in that convention and subsequent documented inadequacies in support provision for Australians with disability, which meant many people, especially those on low incomes, lacked access to the level of funding, services, housing and support required to achieve a decent life [6,7].

In large measure the NDIS does not provide funding for the housing of persons with a disability as the focus is instead on services, for example, access to assistive technologies, worker-provided care and allied therapies, rather than general living assistance [8]. The NDIS includes a small, highly focused housing program—the Specialist Disability Accommodation (SDA) program—developed to encourage investment and growth in disability-appropriate housing supply while also adding to the diversity of accommodation available to people with a disability. However, this program is only intended to meet the housing needs of 6 per cent of persons eligible for NDIS support [8], effectively rationing such assistance to those with the most acute needs. The SDA program does not aspire necessarily to meet the housing needs of persons with a disability, instead it seeks to provide specialist accommodation for those individuals who require a purpose-built home in order to allow them to access NDIS services. The scheme is, therefore, very targeted in its application, and advocates in the disability sector have argued that the high degree of regulation and bureaucratic oversight embedded in the scheme has impeded investment [9], while also leaving the remaining 94 per cent of Australians with a significant disability vulnerable.

In order to understand whether this substantial national policy intervention, the NDIS, has changed the distribution of the risk of homelessness for persons with a disability in Australia, we construct the IRHR using a nationally representative dataset for the periods pre-NDIS rollout and post-NDIS rollout.

## 2. Materials and Methods

In this second paper we explore the changing distribution of the risk of homelessness among populations with a disability, before and after the widespread introduction of the NDIS. Utilizing a large Australian dataset, the Household, Income and Labour Dynamics in Australia (HILDA) Survey, we estimate the risk of homelessness within the Australian population along a 10-point scale from highest relative risk (a score of 10) to lowest relative risk (a score of 1). We compare the homelessness risk for populations with a disability to those without, and across different types of disability.

### 2.1. The Data Set

The HILDA survey is an annual, nationally representative longitudinal study of individuals residing in Australian households. At various times it has been supplemented (to deal with attrition) and it has also been topped up to include recent migrants, making the dataset more representative of the changing nature of the Australian population. The survey has been conducted each year since 2001 and now contains 18 available waves of data [10]. HILDA surveys adults in participating households every year via face-to-face interviews and a self-completion questionnaire, providing detailed data on disability, housing, residential stability and mobility and income alongside broader demographic characteristics. Our previous analysis of homelessness risk focused on the pre-NDIS period (2001–2009). This current analysis compares possible post-NDIS changes in the most recently available data (2017–2018). The HILDA survey dataset consists of approximately 17,000 individuals per wave for waves 1–9 and 23,000 for waves 17–18. Human ethics approval was not required for this research because the analysis used secondary data; that is, the HILDA survey dataset, which is publicly available.

### 2.2. Measuring Disability

There are two ways to measure disability in the HILDA survey dataset. A general question asks whether a respondent has a long-term disability or health condition, to which around 28 per cent of responses across the 18 waves reply in the positive. This question is collected in all waves of the survey. The second variable measuring disability, asked from wave 2 onwards, asks about specific long-term conditions, where the questions are posed in the form, for example, “Which long-term conditions—Hearing problems”. This variable offers comprehensive coverage, including those conditions currently been treated, for example, nervous or emotional conditions, conditions restricting physical activity or work, chronic or reoccurring pain, and long-term effects of a head injury, stroke or other brain damage. We use both measures in the following analyses. The maximum sample size for these measures depends on the pattern and treatment of missing data for the five variables that are used to construct the IRHR (described below).

### 2.3. The Index of Relative Homelessness Risk

The IRHR builds upon previous work on housing precariousness by Mallett et al. [11]. A composite index, the IRHR was first used as a measure of the risk of homelessness in 2012 [12] and then in 2019 for Part 1 of this present work [4]. It is constructed as a simple aggregate of the risk components (detailed in Table 1), which are combined and rescaled to form an index with range from low (1) to high relative risk (10). The index is measured for each individual in the panel dataset, which are then averaged across various groups. In addition, we calculate the relative risk for individuals by major disability types. To examine the data, we use both informal (e.g., descriptive statistics and figures) and formal (paired, or unpaired as appropriate, sample *t*-tests for equality of means, with the Bonferroni adjusted *p*-value for multiple comparisons).

## 3. Results

### 3.1. Pre-NDIS and 2017–18 Index of Relative Homelessness Risk

**Figure 1:**
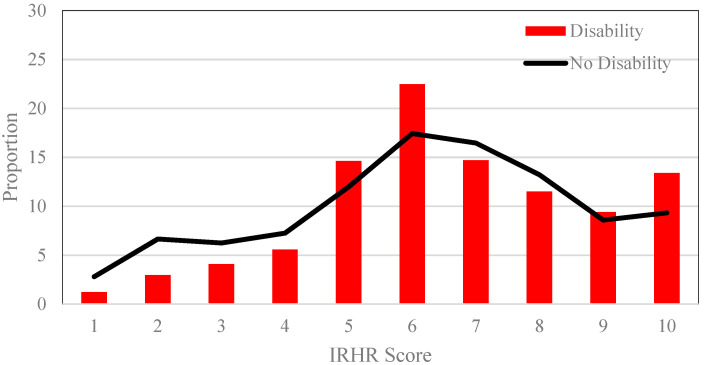
Index of Relative Homelessness Risk by all disability and long-term conditions versus no impairment (post-National Disability Insurance Scheme (NDIS)).

**Figure 2:**
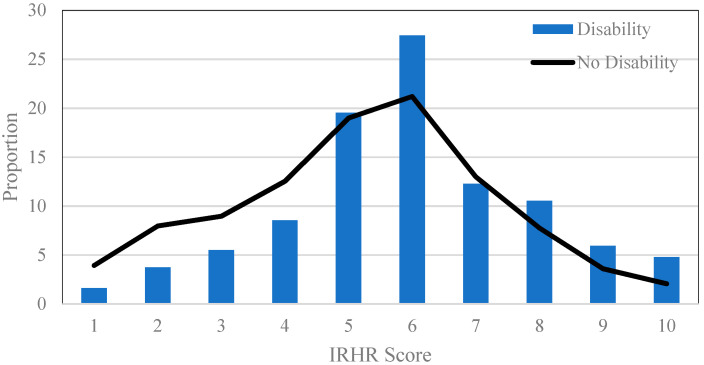
Index of Relative Homelessness Risk by all disability and long-term conditions versus no impairment (pre-NDIS).

In this first part of this analysis we calculate the relative risk of homelessness for all individuals, comparing outcomes in the post-NDIS environment for those with and without a disability (Figure 1). While showing broad similarities in the patterning of risk across those with and without disabilities, there are critical differences. For Figure 1 and Figure 2, there is a statistical difference in the mean value of the IRHR between those with and without a disability both pre-NDIS (*p*-value < 0.000) and post-NDIS (*p*-value < 0.000) (using unpaired two-sample *t*-tests). Compared to unaffected people, those with a disability are on average, significantly over represented in the very high risk category (10) and the mid-risk categories (5 and 6), but under-represented in the very low-risk categories (1, 2, 3, 4). This post-NDIS patterning is notably different to the pre-NDIS outcomes shown in Figure 2. Pre-NDIS, the pattern of difference between the relative risk of homelessness for people with, and without, disabilities were less stark. In this earlier period, people with disabilities were less likely to be at low risk of homelessness (categories 1, 2, 3, 4), and more likely to be at high risk of homelessness (categories 5, 6, 7, 8, 9, 10). These differences in the relative risk of homelessness before and after the implementation of a population-wide policy intervention suggest the benefits of the NDIS may have been distributed unevenly.

### 3.2. The Index of Relative Homelessness Risk by Disability Type

**Figure 3:**
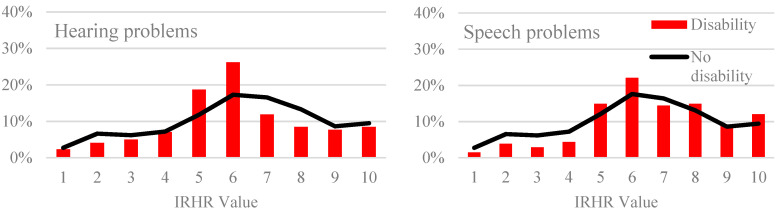

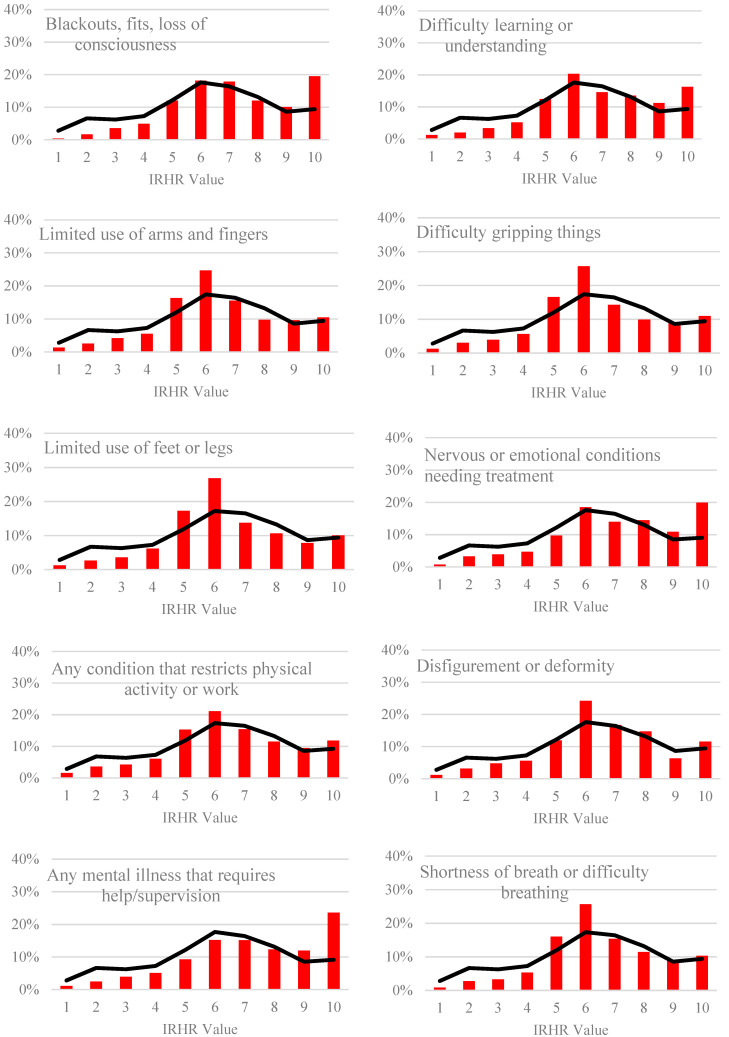

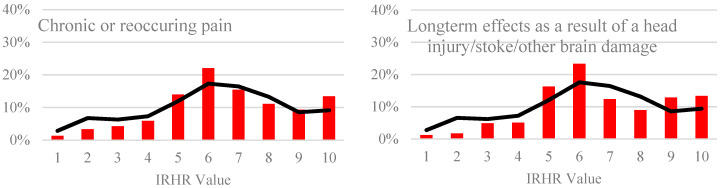
Index of Relative Homelessness Risk by selected disability types (vertical column) versus without impairment (black line).

In order to explore the potential for uneven risk across persons living with different disability types, we examined outcomes at a finer grain by considering specific disabilities. Figure 3 compares the homelessness risk profiles for people with and without a series of key disabilities and chronic conditions, in the post-NDIS period. Similar to the generalised patterns shown in Figure 1, Figure 3 highlights an overall pattern of under representation of people with low homelessness risk. Some conditions (such as hearing or speech problems, limited use of arms/fingers or legs/feet difficulty gripping things, or shortness of breath) are associated with an over-representation of people in the moderate (5–6) risk categories, and—perhaps most concerning—a number of disabilities and conditions are associated with an over-representation of people with very high risk of homelessness. Four disability categories in particular exhibit this ‘up-tick’ of high relative homelessness risk: people who experience blackouts, fits or loss of consciousness, difficulty learning or understanding, nervous or emotional conditions, or mental illness, identifiable in the graphs as red columns at IRHR = 10 that extend well beyond the extent of the black line (signifying the population without the condition). People with these disability conditions should be considered as having particular vulnerability to homelessness in the post-NDIS era.

**Figure 4:**
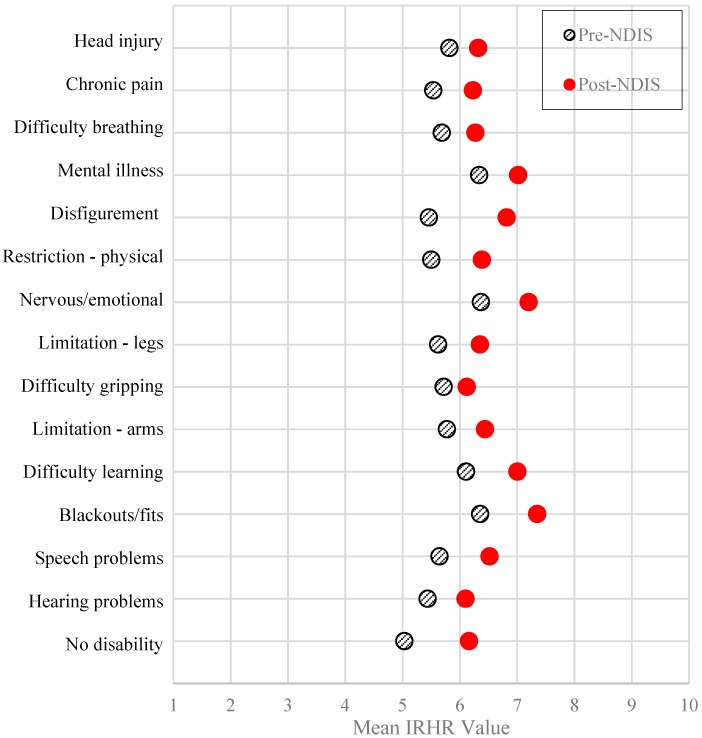
Mean Index of Relative Homelessness Risk Score by selected disability types, pre-NDIS and post-NDIS.

Figure 4 provides a comparison summary of mean homelessness risk by disability type before and after the introduction of the NDIS. It shows an overall increase in mean risk across all disability types, with an especially concerning shift of mean IRHR to above 7 for the four disability types (spanning mental health, learning difficulties and blackouts) identified above. The mean of the IRHR for each disability type pre- and post-NDIS represented in Figure 4 are all statistically significantly different (*p*-value < 0.000).

## 4. Discussion

This analysis has shown that not only do people with disabilities have a different overall pattern of homelessness risk to the wider population, but that some types of disability predispose people to a much higher homelessness risk. A concerning indication of these findings is that, following the introduction of a national scheme broadly designed to improve the lives and living conditions of people with disabilities, homelessness risk does not appear to have improved for this group.

In reflecting on the findings of this analysis it is important to be reminded that homelessness is not a mere part of the housing continuum, it is an outcome where an individual has no suitable housing. Importantly, the effects of homelessness also extend well beyond housing into health, poorer employment (Batterham, 2019) [13], vulnerability to violence (Heerde et al., 2019) [14], and even substantially lower life expectancy (Amato et al., 2019) [15]. For people with disabilities, homelessness is an especially poor housing outcome, likely to amplify their existing health vulnerabilities.

The variation in pattern of risk for different disability types suggests that over and above the baseline risk of homelessness for people with different disabilities and long-term health conditions, the impact of the NDIS has also been uneven. The ‘uptick’ pattern of higher homelessness risk shown in the figures suggests that already at-risk groups (especially people with disabilities affecting cognitive or mental health) within the population with disabilities have missed out on the potential benefits of NDIS, and may be experiencing the double disadvantage of doing so in a policy environment that assumes they have received protection from the NDIS. It is important to acknowledge that when we compare the IRHR pre- and post- the introduction of the NDIS, *no* disability group recorded a fall in the percentage of the group in the highest risk categories. In regard to housing, Figure 3 above appears to suggest that the NDIS unintentionally provides more assistance to those who were least at risk pre-NDIS and, potentially, the least to those in the upper range of the IRHR. Specifically, the figure demonstrates that almost all people with a disability and IRHR score 7+ are more at risk post-NDIS compared to pre-NDIS. We noted at the start of this paper that the NDIS does not specifically target or fund housing and it is not an income support or welfare tool, nor does it provide housing services, and has narrow eligibility. We suggest, then, that the increased relative homelessness risk is an unintended, but serious, consequence.

Finally, we note the limitations of this analysis. Firstly, while Beer et al. [4] include assessment of disability based on the Australian Bureau of Statistics’ (ABS) General Social Survey (GSS), we were not able to replicate that earlier analysis as the ABS last collected the GSS in 2016. Secondly, as the HILDA survey does not cover those residing in non-private dwellings (e.g., nursing homes, transitional housing, hotels or corrective institutions) we are not able to assess outcomes for those individuals who may be accommodated in such forms of housing. Thirdly, although results in the lower and mid-range of the IRHR are mixed, and we note there is no disability type that does not show an increase in the risk of homelessness in 2017 and 2018, this assessment of risk of homelessness does not allow consideration of cause. That is, we cannot attribute the observed poor outcome to the NDIS (we only assess associations), suggesting promising future research opportunities in assessing causality.

## 5. Conclusions

We are cautious in concluding that the introduction of the NDIS has resulted in a higher level of homelessness risk for this vulnerable group within society, as other changes within Australia’s demography, economy and society may have played a part. While no doubt some people with a disability have benefited from the introduction of the NDIS, improvements have been far from widespread. For example, the structural ageing of the population has reduced the number of persons with a disability remaining in the family home and the further contraction of public housing services has reduced opportunities for this group to reside in this tenure. It is notable, however, that the NDIS has clearly *not* reduced the risk of homelessness for persons with a disability in Australia and has certainly not removed this risk. This failure is despite significant public sector outlays on disability services, but service provision alone cannot ensure the quality of life of persons living with an impairment.

It is important to reflect on which disability types appear to be most vulnerable to homelessness in this analysis. People experiencing conditions categorized into four groupings—blackouts, fits or loss of consciousness, difficulty learning or understanding, nervous or emotional conditions, or mental illness—are shown in to have concerning patterns of high relative risk. To some extent, previous work predicts this patterning, suggesting that a combination of exclusion and system complexity may be drivers. Without advocacy and assistance, some people with these types of disabilities are likely to find it difficult to navigate a complex assistance system [16], and some individuals may miss out entirely [17]. The importance of addressing homelessness risk for these groups is all the more important when we consider the very high prevalence of these disability types among NDIS recipients—two thirds of all current recipients, for example, are diagnosed as having autism or some type of intellectual or psychosocial disability, or brain injury [18].

Among ongoing judicial, political, educational and welfare challenges highlighted by the 2019 United Nations Review on the Rights of Persons with a Disability in Australia was the lack of accessible and affordable housing options [19]. The findings of this paper implicate the scale of this unmet need and have clear lessons for public policy. Specifically, there remains an opportunity for the National Disability Insurance Agency (NDIA) to engage more fully with the provision of affordable housing for persons with a disability through the SDA program. A focus on those with the most acute needs overlooks the widespread distribution of unaffordable housing amongst Australians with a disability, and this in turn adds to the risk of homelessness. The reform and expansion of the SDA program represents an essential first step towards better outcomes—with respect to health, financial wellbeing, and security of tenure—for persons living with a disability in Australia.

## Figures and Tables

**Table 1:** Index of Relative Homelessness Risk (IRHR) components from the Household, Income and Labour Dynamics (HILDA) survey dataset (adapted from Beer et al., 2019 [4]).

IRHR Components	HILDA Variables
Cash flow	This variable is a count, at each wave, of the number of cash flow problems that are reported (from a possible 7 in HILDA)
Number of residential moves	A cumulative sum of the number of moves undertaken in the previous wave, the count at wave 1 is zero
Evictions	Variable signifying if the individual was evicted from their last accommodation by the landlord
Low income	Deciles of household income (gross annual income), reverse coded
Housing costs	This variable is constructed using the values for mortgage and rent payments and is structured as deciles of housing cost
